# Robust Manipulation of Randomly Stacked Jenga Blocks via a Strategy-Driven Framework Using a Single RGB-D Sensor †

**DOI:** 10.3390/s26123767

**Published:** 2026-06-12

**Authors:** Dongwoon Song, Yeri Park, Minseong Jo, Wonje Hwang, Gijae Ahn, Seung-Joon Yi

**Affiliations:** Department of Electrical and Electronic Engineering, Pusan National University, Busan 43241, Republic of Korea; dongwoon@pusan.ac.kr (D.S.); yeri@pusan.ac.kr (Y.P.); jominseong@pusan.ac.kr (M.J.); hwj0728@pusan.ac.kr (W.H.); gijaeahn77@pusan.ac.kr (G.A.)

**Keywords:** pick and place, pose estimation, cluttered environment, robot competition

## Abstract

Robust manipulation of small, densely stacked objects remains a challenging problem due to severe occlusions and geometric ambiguities, particularly under single-view sensing conditions. When observed using a single RGB-D sensor, adjacent surfaces of featureless cuboid objects, such as Jenga blocks, often merge in depth measurements, making reliable instance separation and pose estimation difficult. This paper presents a strategy-driven perception and manipulation framework for the robotic rearrangement of randomly stacked Jenga blocks under single RGB-D sensor constraints. The proposed approach employs a heightmap-based perception pipeline that integrates color filtering with geometric reasoning to segment individual blocks and estimate manipulation-compatible poses. Beyond perception, the proposed system determines robot actions through a structured manipulation policy consisting of region-wise search for directly executable grasps, grasp candidate evaluation based on accessibility and collision risk, selective local regrasping for workspace reconfiguration, and placement mode selection between direct insertion and sliding-assisted placement. In this framework, controlled grasp-and-release actions are applied only when no directly executable candidate is found within the currently scanned region and a suitable recovery target can be identified, thereby transforming cluttered local arrangements into more executable states without requiring additional sensing modalities. Experimental results, conducted under competition-equivalent conditions, demonstrate a high task success rate of 99.02%, confirming the robustness and reliability of the proposed framework. The results show that strategy-driven manipulation can effectively compensate for perception limitations in single RGB-D sensor environments, enabling stable and efficient pick-and-place operations in dense clutter.

## 1. Introduction

To expand automation across diverse industrial environments, robust perception and manipulation capabilities are required for objects that are randomly placed or densely stacked within a workspace [1]. In practical scenarios, objects are introduced without prior alignment, and their positions and orientations continuously change during operation. Under such conditions, the ability to reliably distinguish individual objects and accurately estimate their poses is essential for stable robotic manipulation [2].

However, many existing approaches are developed under structured assumptions, such as regular grid arrangements, uniform backgrounds, or fiducial markers [3,4]. While these assumptions simplify system design and improve performance, they significantly limit applicability in unstructured environments where small objects are randomly stacked in multiple layers, often adjacent to or partially overlapping [5].

This limitation becomes particularly critical when handling small cuboid objects, such as Jenga blocks, in densely cluttered and highly occluded multi-layer configurations [6,7]. Under single-view RGB-D sensing, complex spatial arrangements obscure object boundaries, leading to degraded pose estimation accuracy [8]. Furthermore, even when segmentation is successful, the estimated poses are often not directly compatible with downstream manipulation constraints, such as grasp accessibility and strict placement requirements [9,10].

**Figure 1:**
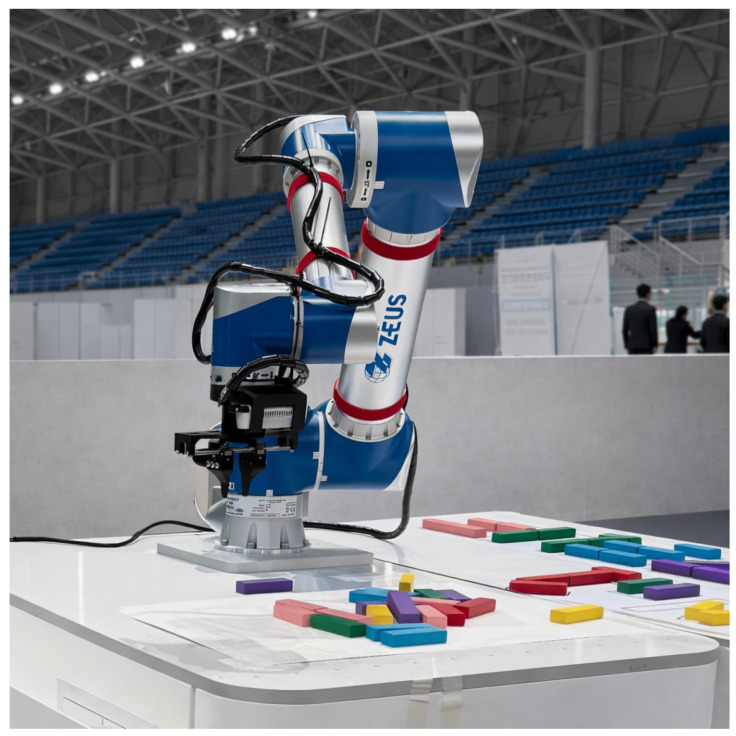
Zeus Robot Mission Challenge 2025.

**Figure 2:**
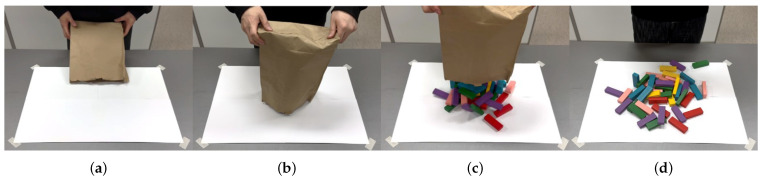
Random placement process of Jenga blocks: (**a**) empty workspace, (**b**) random pouring, (**c**) dense stacked arrangement, and (**d**) final initial condition for task execution.

This study addresses these challenges in the context of the 2025 International Robot Contest (IRC) Zeus Robot Mission Challenge, where the actual competition environment is shown in Figure 1. The task requires rearranging tightly stacked blocks (see Figure 2) according to predefined color patterns using strictly a single Intel RealSense D435 sensor. This single-sensor constraint necessitates a unified framework that tightly couples perception with manipulation-oriented reasoning, rather than treating them as independent modules.

Although prior studies have investigated object segmentation, pose estimation, and robotic grasping in cluttered scenes, the rearrangement of densely stacked Jenga blocks presents a practical gap between visual recognition and executable manipulation. Under a single RGB-D sensor constraint, a detected block is not necessarily graspable because of occlusion, limited gripper clearance, inter-block contact, unstable poses, and strict placement requirements. Therefore, this task requires not only accurate perception but also a manipulation strategy that determines whether, when, and how a detected block can be safely grasped and placed.

Accordingly, we propose a strategy-driven perception and manipulation framework based on a heightmap representation that integrates both color and geometric information. The proposed approach enables robust segmentation of objects in severe multi-layer clutter while generating manipulation-aware pose representations directly compatible with grasp planning. In addition, iterative strategies, including regrasping for vertically oriented blocks and obstacle-aware manipulation, are incorporated to improve task robustness. Ultimately, the framework ensures reliable task execution under strict single-view constraints in real-world competition environments.

A preliminary version of this work has been accepted for presentation and publication at the International Conference on Ubiquitous Robots (URs) 2026 [11]. The conference version presented the basic heightmap-based perception and manipulation framework for rearranging randomly stacked Jenga blocks using a single RGB-D sensor. Compared with the conference version, this article provides a substantially expanded treatment of the strategy-driven manipulation framework, including detailed decision flows, grasp feasibility evaluation, local regrasping and sliding-assisted placement strategies, implementation parameters, additional experimental analysis, supplementary comparison under different clutter conditions, and a discussion of limitations and generalization.

## 2. Related Work

In visually guided robotic grasping, particularly under single-view constraints, heightmap representations have been widely adopted due to their direct compatibility with planar manipulation strategies [12,13]. Early approaches based on heightmaps effectively isolate objects using depth discontinuities or color thresholding, assuming sufficient spatial separation between items [14,15]. However, in densely stacked and overlapping configurations, occlusions introduce geometric ambiguities in the heightmap, making purely heuristic-based separation unreliable [16].

To address complex cluttered scenes, recent studies have increasingly employed deep learning frameworks for instance segmentation and 6D pose estimation [17,18]. These approaches demonstrate strong performance in general bin-picking tasks; however, their applicability becomes limited when dealing with featureless, identical cuboid objects tightly packed in multi-layer configurations. In such cases, distinguishing boundaries between coplanar adjacent blocks remains challenging, often resulting in merged instances and inaccurate depth predictions [19]. Moreover, data-driven methods typically require large-scale domain-specific datasets and multi-view sensing setups [7], which are difficult to satisfy under strict single-view hardware constraints.

Beyond the methodological differences between heuristic and learning-based approaches, many existing perception pipelines primarily focus on object recognition while treating manipulation as a separate downstream process. Although these methods provide accurate pose estimates, they often do not explicitly consider whether the estimated poses are feasible for grasp execution without collision in cluttered environments. For tasks involving densely stacked objects, such as rearranging randomly placed Jenga blocks, integrating perception with manipulation constraints becomes essential.

To address this limitation, the proposed framework extends the conventional heightmap-based approach by tightly integrating color and geometric information for robust object separation under severe occlusions. Furthermore, the perception process is explicitly formulated in a manipulation-aware manner, enabling direct generation of grasp-compatible pose representations and facilitating collision-free grasp selection under single-view constraints.

## 3. System Overview

The proposed framework is implemented on a robotic manipulation platform deployed in the 2025 International Robot Contest (IRC) Zeus Robot Mission Challenge environment. The system consists of a robotic manipulator equipped with an Intel RealSense D435 RGB-D sensor and a custom-designed linear parallel gripper. In accordance with the competition constraints, all perception and planning processes rely exclusively on RGB-D data acquired from the single sensor.

Although the competition allowed flexible camera placement, the RGB-D sensor was deliberately mounted on the robot wrist instead of a fixed overhead position. This design choice enables active viewpoint control during task execution, allowing the system to acquire localized observations by repositioning the end-effector. Rather than relying on a global static view, the workspace is incrementally explored through sequential scanning of partitioned subregions. In practice, the robot observes each local region in a top-to-bottom manner. Starting from the upper part of a selected subregion, the wrist-mounted sensor progressively scans downward while evaluating whether any detected block satisfies the conditions for direct grasp execution. When such a block is found, the robot immediately performs the grasping action and then returns to the same local region to resume scanning of the remaining lower area. If no directly executable block is found within the current region, the system attempts local regrasping to physically reconfigure the nearby workspace and improve grasp accessibility. After this local recovery step, the same region is rescanned once more before the robot shifts laterally to a neighboring subregion and repeats the same top-to-bottom scanning process. When no suitable recovery target can be identified within the current region, the system skips forced intervention and proceeds directly to the next region. This procedure allows the system to maintain local perception continuity while incrementally covering the entire cluttered workspace under single-sensor constraints.

This observation strategy provides two key advantages. First, localized sensing reduces depth ambiguity in densely stacked regions by focusing on smaller areas with improved geometric consistency. Second, the perception system remains fully integrated within the robot kinematic chain, simplifying coordinate transformation and spatial reasoning between sensing and manipulation. Notably, the framework operates entirely under single-sensor constraints without requiring multi-view fusion or external calibration structures.

**Figure 3:**
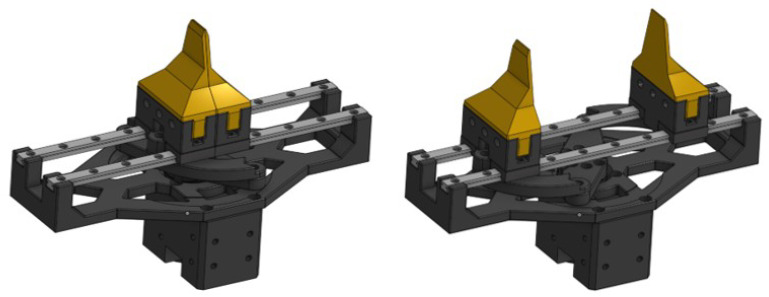
Custom-designed linear parallel gripper.

The custom linear parallel gripper, as shown in Figure 3, is designed to stably grasp Jenga blocks along both their major and minor axes. Its stroke range and finger geometry enable flexible grasp configurations depending on local accessibility. This mechanical design directly influences the perception module, as the estimated pose parameters are required to align with feasible grasp directions in both orientations. Consequently, pose estimation is formulated in a manipulation-aware manner rather than as a purely geometric computation.

At the system level, perception and motion planning are tightly integrated within a unified framework. The estimated pose parameters are directly evaluated in terms of grasp accessibility, collision risk with neighboring objects, and required reorientation strategies. This tight coupling enables seamless transition from perception to action, ensuring reliable task execution under strict single-view constraints in a real-world competition environment.

## 4. Perception Method

**Figure 4:**
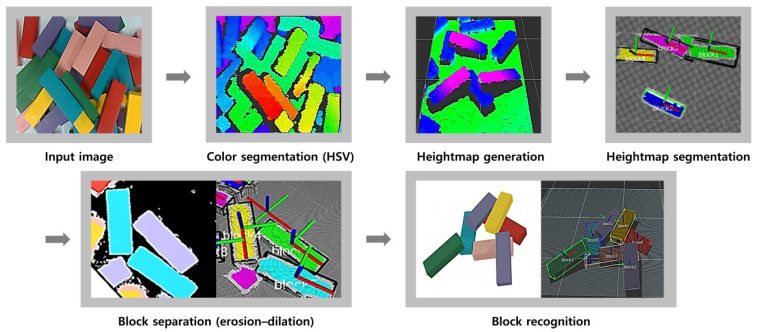
Perception pipeline for randomly placed Jenga blocks, including HSV-based color segmentation, heightmap generation, heightmap segmentation, morphological block separation, and block recognition.

The overall perception pipeline for randomly placed Jenga blocks is illustrated in Figure 4.

### 4.1. RGB-D to Workspace Representation

The perception process begins by transforming raw RGB-D data into a geometrically consistent workspace representation. For a depth pixel at image coordinate (u,v) with depth value d(u,v), the corresponding 3D point in the camera coordinate frame is computed as

(1) $$p_{c}=d\left(u,v\right)K^{-1}{\left[u,v,1\right]}^{T},$$

where $p_{c}$ denotes the 3D point in the camera coordinate frame, K is the intrinsic calibration matrix of the RGB-D sensor, and ${\left[u,v,1\right]}^{T}$ is the homogeneous pixel coordinate.

The computed point is then transformed into the robot base frame through a rigid transformation

(2) $$p_{b}=Rp_{c}+t,$$

where R and t represent the extrinsic calibration between the wrist-mounted sensor and the robot base.

Instead of directly processing the full 3D point cloud, the proposed framework constructs a heightmap representation by projecting the transformed points onto the planar workspace. For a discretized planar grid $\left(x_{i},y_{j}\right)$, the height value is defined as

(3) $$H\left(x_{i},y_{j}\right)=\max_{\left(x,y,z\right)\in C_{ij}}z,$$

where $H(x_{i},y_{j})$ denotes the height value of the grid cell at $\left(x_{i},y_{j}\right)$, and $C_{ij}$ represents the set of transformed 3D points projected onto the corresponding grid cell.

This 2.5D representation converts complex multi-layer clutter into a structured surface model while preserving geometric information relevant for planar manipulation. By reducing the dimensionality of the raw point cloud, the heightmap formulation enables efficient and robust processing under single-view sensing constraints.

### 4.2. Color-Guided Candidate Extraction

To suppress background interference and improve robustness under cluttered conditions, RGB data are transformed into the HSV color space. Object regions are identified using predefined color similarity thresholds corresponding to the block colors used in the competition. The extracted regions serve as spatial priors that constrain the subsequent heightmap-based segmentation process.

By integrating color filtering with geometric reasoning, the framework effectively reduces false detections caused by shadows, illumination variations, and depth noise, thereby improving the reliability of candidate extraction.

### 4.3. Heightmap-Based Block Segmentation

Segmentation is performed by exploiting height discontinuities in the constructed heightmap representation. In multi-layer stacking scenarios, adjacent blocks typically exhibit small but consistent vertical differences, which can be detected through local gradient analysis with predefined thresholds.

However, when adjacent blocks have similar height values and appear merged in the heightmap, additional refinement is required. To address this, morphological operations are applied to separate weakly connected regions and recover object boundaries. Specifically, erosion is used to disconnect adjacent regions, followed by dilation to restore the geometric extent of individual blocks.

This approach enables reliable separation of partially overlapping cuboid objects without requiring multi-view reconstruction, making it well-suited for operation under single-view sensing constraints.

### 4.4. Pose Estimation via Principal Axis Analysis

For each segmented region, planar pose estimation is performed using principal component analysis (PCA). Given a set of inlier points $\left\{q_{k}\right\}$ belonging to a segmented block projected onto the planar surface, the covariance matrix is computed as

(4) $$\Sigma =\frac{1}{N}\sum_{k=1}^{N}\left(q_{k}-\bar{q}\right){\left(q_{k}-\bar{q}\right)}^{T},$$

where $\bar{q}$ denotes the centroid of the region.

The eigenvectors of Σ define the principal axes of the block footprint, while the corresponding eigenvalues indicate the spatial extent along each axis. The dominant eigenvector determines the planar orientation used for grasp direction selection, enabling the gripper to align with both aligned and slightly tilted blocks.

To improve robustness against false detections, geometric consistency checks are applied based on expected block dimensions, aspect ratio, and color density. Only regions satisfying these constraints are retained as valid grasp candidates for subsequent manipulation.

### 4.5. Manipulation-Aware Output Representation

Unlike purely geometric pose estimation frameworks, the extracted parameters are structured to support manipulation. Each detected block is represented by its centroid, orientation, major and minor axis lengths, and surrounding free-space margin. The free-space margin is computed from neighboring heightmap cells to estimate collision risk during grasp approach.

This representation enables grasp feasibility evaluation and motion planning without additional geometric processing. The estimated poses can be directly used for collision-aware manipulation.

## 5. Strategy-Driven Manipulation Under Single-View Constraints

### 5.1. Manipulation-Oriented Decision Flow

The proposed framework determines robot actions through a manipulation-oriented decision flow rather than by directly executing perception results as fixed commands. Under single-view RGB-D constraints, the estimated pose of a detected block does not always imply that the block is immediately executable for grasping and placement. Therefore, each detected block is interpreted as a manipulation candidate and is further evaluated in terms of accessibility, collision risk, and compatibility with the target placement condition.

As summarized in Algorithm 1, the workspace is partitioned into nine local regions, which are sequentially scanned using the wrist-mounted RGB-D sensor. For each region, the system first searches for directly executable grasp candidates based on the estimated pose and surrounding free-space condition. If a feasible candidate is found, the robot immediately performs grasping and placement. Otherwise, the system attempts local regrasping within the currently scanned region in order to physically reconfigure the nearby workspace and improve grasp accessibility. After this local recovery action, the same region is rescanned and the search for executable candidates is repeated before the robot proceeds to the next region. If no suitable recovery target is identified, the system does not force regrasping; instead, it defers intervention in that region and moves on to the next region for continued exploration. In this sense, regrasping is treated not as a mandatory fallback action, but as a selective region-wise recovery strategy tightly coupled with local perception and action.

Under single-view RGB-D sensing, the perceived block state may differ from the actual physical arrangement when severe occlusion, missing depth values, weak color contrast, or nearly coplanar contact between adjacent blocks occurs. In particular, tightly contacting blocks with similar height values may be merged in the heightmap, while partially hidden blocks may not provide sufficient geometric evidence for reliable pose estimation. To reduce the effect of such perception-reality mismatch, the proposed framework applies geometric consistency checks and evaluates each detected candidate in terms of grasp accessibility and collision risk before execution. Uncertain or poorly accessible candidates are therefore rejected during grasp evaluation, and the workspace is rescanned after local regrasping when physical reconfiguration is required.
**Algorithm 1:** Decision Flow of the Proposed Manipulation Framework
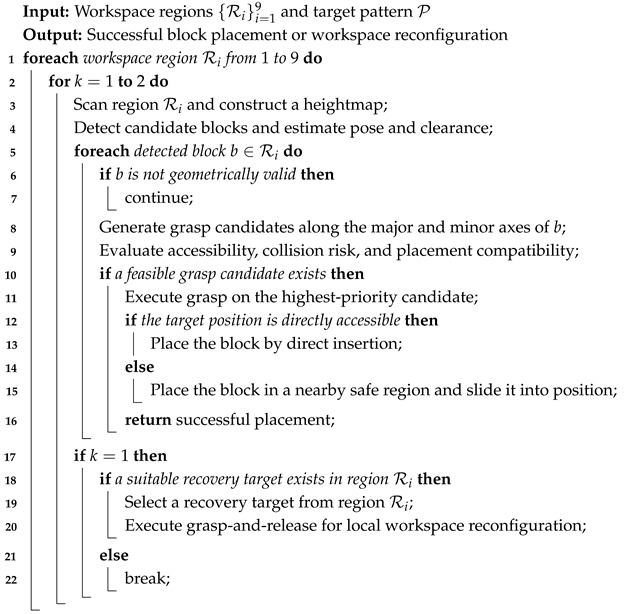


### 5.2. Region-Wise Search and Grasp Candidate Evaluation

For each scanned local region, the perception module provides the centroid, planar orientation, principal axes, and local free-space margin of each detected block. Based on this information, the manipulation module generates grasp candidates along both the major and minor axes of the block. This formulation is motivated by the mechanical capability of the custom linear parallel gripper, which can stably grasp Jenga blocks from either their long side or short side depending on local accessibility.

**Figure 5:**
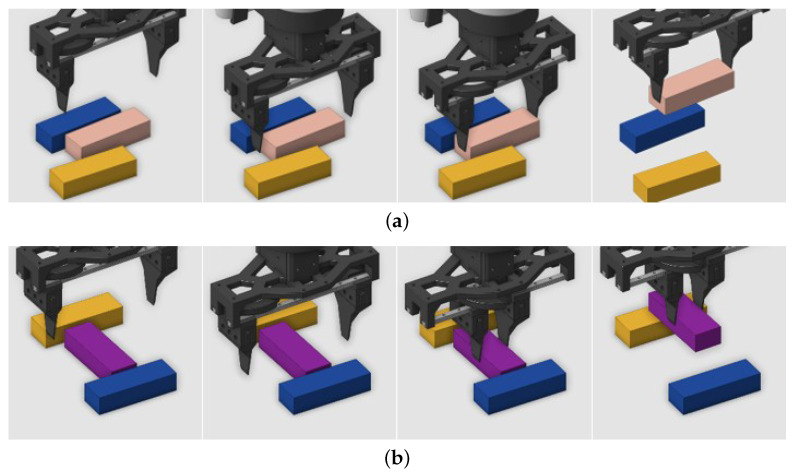
Block grasping strategies: (**a**) grasping along the major axis of a block; (**b**) grasping along the minor axis of a block.

Each grasp candidate is evaluated according to approach clearance, surrounding free-space margin, collision risk with neighboring blocks, and compatibility with the desired placement orientation. Among the feasible candidates, the robot selects the one that maximizes direct executability while minimizing the likelihood of interference during approach and subsequent placement. Representative examples of grasp execution along different grasp directions are shown in Figure 5. Through this process, the system prioritizes direct grasping whenever the local block state and placement requirement are jointly satisfied. The candidate ranking follows a conservative priority rule. First, collision-prone or poorly accessible candidates are rejected. Next, candidates that are directly compatible with the desired placement orientation are prioritized over those that would require additional recovery or corrective manipulation.

A detected block is considered non-executable for direct grasping when sufficient gripper clearance is not available, neighboring blocks obstruct the finger insertion path, the block is in an unstable edge-standing configuration, or the segmented region fails the geometric consistency checks required for reliable pose estimation. In these cases, the framework does not force direct execution; instead, it selects another feasible candidate or performs local regrasping followed by rescanning to recover a workspace state with improved grasp accessibility.

To improve reproducibility, the main implementation parameters used for color segmentation, geometric validation, and grasp feasibility evaluation are summarized in Table 1.

### 5.3. Handling Tilted but Directly Graspable Blocks

Not all blocks observed in the workspace lie in perfectly aligned and stable planar poses. In practice, some blocks appear with noticeable local inclination due to uneven support from neighboring objects. However, such tilted configurations do not always require immediate regrasping. When sufficient approach clearance is available, the proposed system directly grasps these blocks by aligning the gripper with the estimated major or minor axis of the inclined footprint.

**Figure 6:**
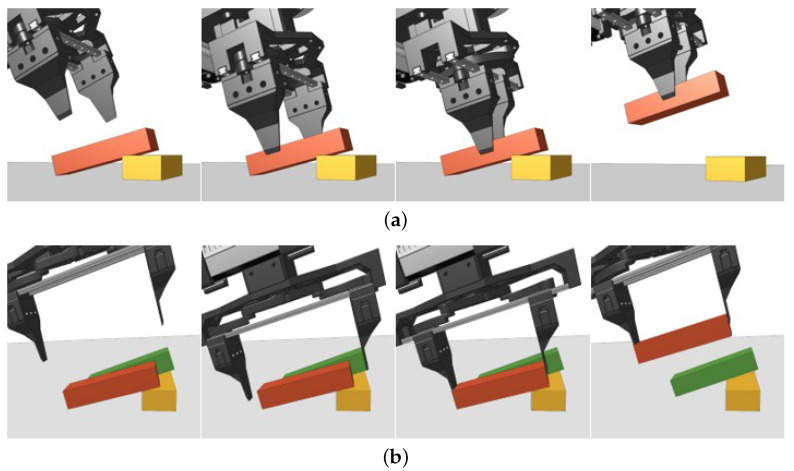
Tilted Tilted block grasping strategies: (**a**) direct grasping of a tilted block along its short side; (**b**) direct grasping of a tilted block along its long side.

This strategy is particularly useful because it prevents unnecessary recovery actions for blocks that are geometrically tilted but still executable for grasping. As illustrated in Figure 6, the robot can accommodate tilted configurations by selecting either long-side or short-side grasping depending on the local arrangement. In this way, the system distinguishes between blocks that are merely tilted yet graspable and those that require physical reconfiguration before reliable manipulation can proceed. 

### 5.4. Local Regrasping for Workspace Reconfiguration

When no directly executable grasp candidate is found within the currently scanned local region, the system attempts local regrasping for workspace reconfiguration. This strategy is not intended as a simple retry of the same grasp attempt, but as a deliberate manipulation step to transform the local workspace into a more executable state. In the proposed framework, regrasping is therefore treated as a selective region-wise recovery policy that is activated only when direct execution is not available within the current region. To clarify how recovery actions are initiated under this condition, the prioritization of recovery targets is summarized in Algorithm 2.
**Algorithm 2:** Recovery Target Prioritization Within a Local Region
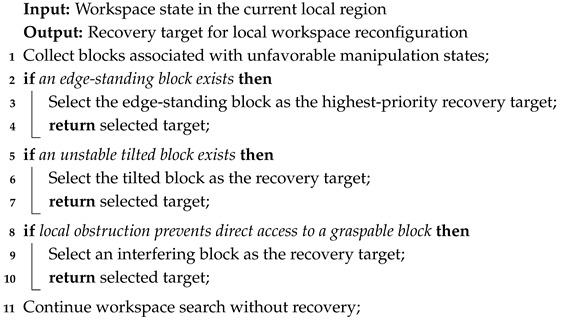


**Figure 7:**
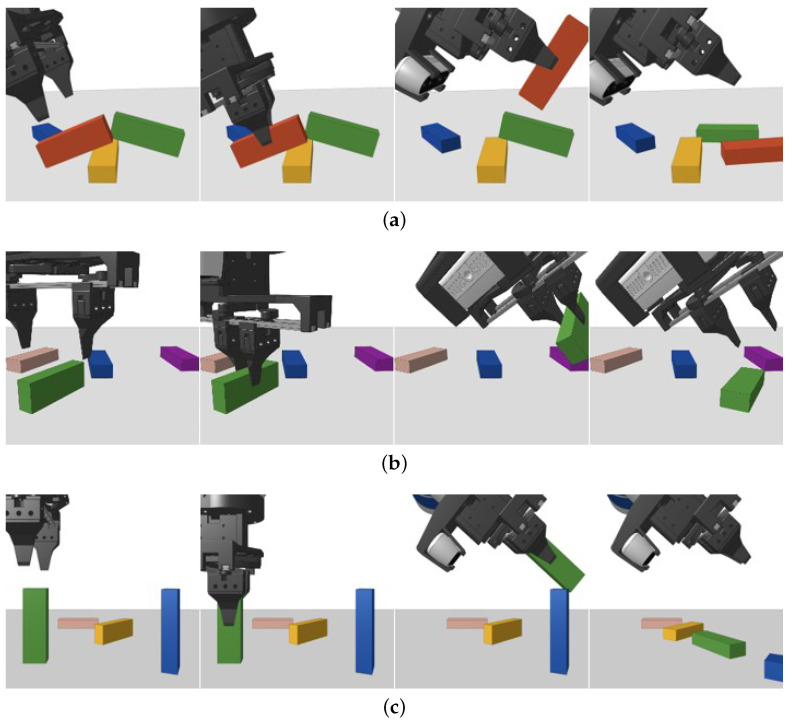
Block regrasping strategies: (**a**) regrasping of an unstable tilted block for local reconfiguration; (**b**) recovery of an unstable upright block by controlled grasp-and-release; (**c**) recovery of an edge-standing block by controlled grasp-and-release.

Typical recovery targets include edge-standing blocks, unstable blocks supported on narrow contact surfaces, and interfering blocks that prevent direct access to otherwise graspable candidates. In these cases, the robot selects an appropriate recovery target and performs a controlled grasp-and-release action to induce gravitational reorientation or reduce local occlusion. The resulting physical rearrangement often improves the resting stability of the manipulated block and increases the accessibility of neighboring blocks. After each recovery action, the same region is rescanned and the search for executable candidates is repeated. If no suitable recovery target can be identified within the current region, the system does not force regrasping; instead, it skips intervention in that region and proceeds to the next region for continued exploration. Representative examples of such recovery behaviors are shown in Figure 7. Through this iterative scan–search–reconfigure loop, the proposed framework compensates for the limitations of single-view sensing without requiring additional sensing modalities.

### 5.5. Placement Mode Selection Under Target Constraints

After a block has been successfully grasped, the final placement action is selected according to the accessibility of the target position in the template. When sufficient clearance exists around the target site, the robot performs direct insertion by lowering the block vertically into the designated position. This mode is efficient and is preferred whenever the surrounding arrangement allows collision-free approach.

**Figure 8:**
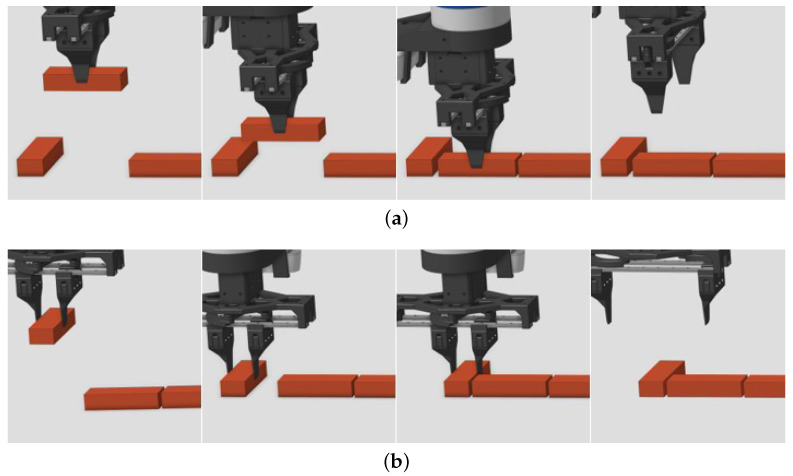
Block placement strategies: (**a**) direct insertion into the target position; (**b**) sliding-assisted insertion, in which the block is first placed near the target and then pushed laterally into the final position.

However, when the target region is tightly constrained by already placed neighboring blocks, direct insertion becomes prone to mechanical interference. To address this problem, the proposed framework employs a sliding-based placement mode. In this mode, the robot first lowers the grasped block into a nearby collision-safe region and subsequently applies a lateral sliding motion to move the block into its final target position. As shown in Figure 8, this strategy enables accurate placement while reducing the risk of overlap or contact-induced misalignment in dense template configurations. Accordingly, placement is treated not as a single fixed motion primitive, but as a mode selection problem between direct insertion and sliding-assisted insertion.

## 6. Experiment

### 6.1. Evaluation Protocol

The proposed system was evaluated over five independent execution runs conducted under the same sensing and task constraints as the IRC Zeus Robot Mission Challenge. In each run, the target template required the placement of 41 Jenga blocks, and the robot repeatedly executed the full perception-manipulation pipeline until all required placements had been attempted.

The initial block arrangement for each run was generated using the same random placement procedure described earlier in the paper, where the blocks were poured and distributed randomly in the workspace to form dense, cluttered, and partially stacked configurations. All evaluations were performed using the wrist-mounted Intel RealSense D435 RGB-D sensor (Intel Corporation, Santa Clara, CA, USA) and the complete perception-manipulation framework described in Section 3, Section 4 and Section 5.

Rather than isolating individual perception components under simplified laboratory conditions, the evaluation was designed to reflect the actual deployment scenario, in which perception and manipulation must operate jointly in cluttered multi-layer environments under strict single-sensor constraints.

### 6.2. Performance Metrics

Performance was assessed using task-level metrics that directly reflect practical manipulation reliability. Each placement attempt was evaluated individually and was regarded as failed if the placed block either deviated from the designated template region or overlapped with another block already placed on the template. The primary evaluation metrics were the task success rate, the average completion time per successful placement, and the number of regrasping actions recorded in each run. The task success rate was defined as the ratio of successful placements to total placement attempts. The completion time was measured from the beginning of the local scan associated with the selected target block to the completion of the final placement motion. A regrasping action was counted when the robot intentionally grasped and released an interfering or unstable block to induce local reconfiguration before resuming grasp feasibility evaluation. For clarity, the regrasping actions reported in Table 2 refer to the total number of such grasp-and-release operations, whereas the regrasp-based placements analyzed in Table 3 refer to the number of finally placed blocks that required at least one regrasping step. For summary reporting, both quantities were additionally averaged across the five execution runs. In addition to the main task-consistent evaluation, a supplementary comparison was conducted over five trials for each condition to examine the effects of clutter level and the use of regrasping under representative workspace settings. The compared conditions consisted of a fully spread arrangement without regrasping, a random clutter arrangement without regrasping, and a random clutter arrangement with regrasping.

### 6.3. Experimental Results

As summarized in Table 2, the proposed framework achieved a mean task success rate of 99.02% across the recorded execution runs, with an average completion time of 4.11 s per successful placement. The average number of regrasping actions per trial was 7.80.

**Figure 9:**
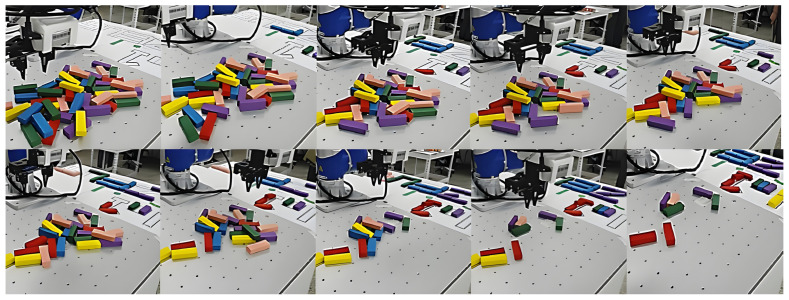
Temporal progression of randomly stacked Jenga blocks.

**Figure 10:**
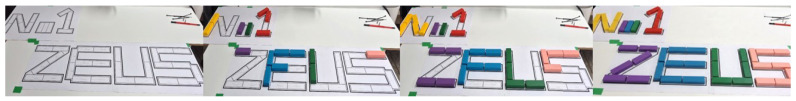
Temporal progression of block placement on the template.

These results indicate that the iterative regrasping strategy was frequently activated in densely stacked configurations, confirming its role as an essential component of the manipulation pipeline rather than a rare recovery mechanism. Representative execution sequences are shown in Figure 9 and Figure 10, illustrating progressive workspace reconfiguration, improved grasp feasibility, and stable final placement under the given pattern constraints. Representative execution videos are also provided online to show the complete picking, regrasping, and placing processes (https://www.youtube.com/watch?v=VY-aq4uh1sQ, accessed on 20 May 2026).

To further analyze the role of regrasping, Table 3 categorizes regrasp-based placements according to the representative unfavorable block states considered in the proposed manipulation strategy. The results show that edge-standing cases accounted for most of the regrasp-based placements, whereas tilted block cases were observed only rarely.

A supplementary comparison under different workspace conditions and strategy settings is discussed in the next subsection to further examine the practical role of regrasping.

### 6.4. Discussion

The evaluation results demonstrate that the proposed heightmap-based perception framework, when tightly integrated with task-aware manipulation strategies, can reliably operate under strict single-sensor constraints in cluttered multi-layer environments. Although component-level comparisons with alternative segmentation methods are not provided, task-consistent validation under realistic operational conditions offers strong evidence of system-level robustness.

A supplementary comparison under different workspace conditions further clarifies the role of regrasping in the proposed framework. Although this comparison is not intended as a direct benchmark against external bin-picking systems, it serves as an ablation-style evaluation of the proposed regrasping strategy under different clutter conditions. As summarized in Table 4, direct grasping without regrasping already achieved a high mean success rate of 96.34% when the blocks were fully spread and spatially separated, indicating that the perception and direct manipulation pipeline itself is effective when severe inter-block interference is absent. In contrast, when the same grasping framework was applied to randomly cluttered arrangements without regrasping, the mean success rate dropped to 78.86%, showing that dense overlap and close adjacency substantially reduce direct executability. When regrasping was enabled under the same random clutter condition, the mean success rate recovered to 99.02%, which is close to complete task execution. These observations suggest that regrasping is not uniformly necessary across all workspace conditions, but becomes particularly important in dense clutter, where local physical reconfiguration is required to restore grasp accessibility and stable placement.

The detailed regrasping analysis in the main evaluation further suggests that the main practical difficulty in the recorded runs was not locally inclined block poses themselves, but rather unstable configurations supported on a narrow face or edge. One possible interpretation is that locally tilted blocks were less likely to persist as dominant failure-inducing states during random block placement. In addition, some tilted configurations may have been naturally converted into more stable and directly manageable states through intermediate regrasping steps. This tendency indicates that the proposed strategy was particularly effective in resolving the more persistent edge-standing cases that directly interfered with stable placement.

Overall, these findings highlight the effectiveness of combining geometric reasoning with iterative physical reconfiguration, enabling initially unfavorable block arrangements to be progressively transformed into manipulation-ready states.

Nevertheless, the present evaluation has several limitations. The number of full task-level execution runs was limited to five because each run required complete rearrangement under competition-equivalent conditions. Therefore, the reported results should be interpreted as task-consistent validation rather than exhaustive statistical benchmarking. In addition, the current implementation assumes colored cuboid objects with approximately known dimensions, which is consistent with the target competition task but limits direct generalization to arbitrary object categories. Future work will include larger-scale trials under controlled clutter levels and extensions to cuboid objects with more diverse appearances and dimensions.

## 7. Conclusions

In this paper, we presented a strategy-driven perception and manipulation framework for the robotic rearrangement of randomly stacked Jenga blocks using a single RGB-D sensor. The proposed perception pipeline combines color-guided candidate extraction with geometric reasoning on a workspace heightmap to segment densely stacked blocks and estimate manipulation-relevant pose parameters, including planar orientation, principal axes, and local free-space conditions.

Beyond perception, the main contribution of this work lies in the design of a manipulation policy that remains effective under inherently incomplete single-view observations. Rather than attempting to eliminate all perception ambiguity before action, the proposed system resolves actionability through region-wise search for directly executable grasps, grasp candidate evaluation based on accessibility and collision risk, selective local regrasping for workspace reconfiguration, and mode selection between direct insertion and sliding-assisted placement. This manipulation-oriented strategy enables the robot to convert uncertain and cluttered observations into reliable task execution under strict single-sensor constraints.

Task-consistent experiments conducted under competition-equivalent conditions demonstrated that the proposed framework can reliably rearrange densely stacked cuboid objects with a high success rate. The results further indicate that controlled grasp-and-release actions are not merely recovery operations, but an essential component of the overall manipulation strategy for improving grasp accessibility and resolving unfavorable block states. In particular, the analysis suggests that the framework is especially effective in handling persistent edge-standing configurations that directly interfere with stable placement. Supplementary comparisons further indicate that direct grasping alone is often sufficient when blocks are well separated, whereas selective local regrasping becomes particularly important under dense clutter, where physical reconfiguration is required to recover executability.

Overall, the proposed framework shows that robust manipulation in dense clutter can be achieved not only through geometric perception, but also through structured coupling between perception and action. Future work will extend the framework to a broader range of cuboid objects with varying appearances and material properties, and will include more systematic benchmarking under controlled clutter levels to further evaluate robustness and generalization.

## Figures and Tables

**Table 1:** Main implementation parameters used for perception and grasp feasibility evaluation.

Parameter	Value	Purpose
Hue/saturation thresholds	20/60	Color similarity filtering
Minimum value threshold	30	Background suppression
Color distance threshold	1.2	Color label assignment
Morphological filter parameters	{3, 2, 18}	Block separation
Side-grasp clearance threshold	24 mm	Side-grasp feasibility
Long-grasp clearance threshold	50 mm	Long-grasp feasibility
Early-scan clearance thresholds	28 mm/56 mm	Conservative initial filtering
Minimum valid region size	67 mm × 17 mm	False-positive rejection
Maximum minor-axis size	20 mm	Geometric consistency check
Minimum fill rate	0.70	Region validity check
Recovery-candidate fill rate	0.57	Recovery target validation
Vertical block height threshold	60 mm	Edge-standing block detection
Initial-scan pitch threshold	5°	Tilted long-grasp rejection
Workspace lateral bound	±0.073 m	Region validity check

**Table 2:** Overall task performance and total regrasping actions in the robotic rearrangement of randomly stacked Jenga blocks.

Test	Number of Jenga Blocks	Success Rate (%)	Avg. Time (s)	Total Number of Regrasping Actions
1	41	97.56 (40/41)	4.07	5
2	41	100 (41/41)	4.05	3
3	41	97.56 (40/41)	4.13	8
4	41	100 (41/41)	4.14	9
5	41	100 (41/41)	4.15	14
**Overall Mean**	–	**99.02**	**4.11**	**7.8**

**Table 3:** Analysis of placements requiring regrasping in the robotic rearrangement of randomly stacked Jenga blocks.

	Placement		Regrasp Cases	
Test	Direct	Regrasp	Tilted Block	Edge Standing
1	36	4	0	4
2	38	3	0	3
3	35	5	0	5
4	34	7	1	6
5	28	13	1	12
**Overall Mean**	**34.2**	**6.4**	**0.4**	**6.0**

**Table 4:** Supplementary comparison under different workspace conditions and strategy settings, evaluated over five trials for each condition.

Condition	Regrasping	Mean Success Rate (%)	Mean Completion Time (s)
Spread	No	96.34	5.26
Random clutter	No	78.86	4.61
Random clutter	Yes	99.02	4.11

## Data Availability

Dataset available on request from the authors.

## References

[1] Yoshida R., Tasaki T. (2025). Pose Estimation Focusing on One Object Based on Grasping Quality in Bin Picking. J. Robot. Mechatron..

[2] Periyasamy A.S., Schwarz M., Behnke S. Robust 6D Object Pose Estimation in Cluttered Scenes Using Semantic Segmentation and Pose Regression Networks. Proceedings of the 2018 IEEE/RSJ International Conference on Intelligent Robots and Systems (IROS).

[3] Oščádal P., Heczko D., Vysocký A., Mlotek J., Novák P., Virgala I., Sukop M., Bobovský Z. (2020). Improved Pose Estimation of Aruco Tags Using a Novel 3D Placement Strategy. Sensors.

[4] Asaoka T., Nagata K., Nishi T., Mizuuchi I. (2018). Detection of object arrangement patterns using images for robot picking. Robomech J..

[5] Park T., Bae G., Shin W., Mammadov M., Seo J., Shin H., Oh H. (2026). PACMAN: Rapid identification of keypoint patch-based fiducial marker in occluded environments. Image Vis. Comput..

[6] Yang J., Li D., Waslander S.L. (2021). Probabilistic Multi-View Fusion of Active Stereo Depth Maps for Robotic Bin-Picking. IEEE Robot. Autom. Lett..

[7] Duffhauss F., Demmler T., Neumann G. MV6D: Multi-View 6D Pose Estimation on RGB-D Frames Using a Deep Point-wise Voting Network. Proceedings of the 2022 IEEE/RSJ International Conference on Intelligent Robots and Systems (IROS).

[8] Jafari O.H., Mustikovela S.K., Pertsch K., Brachmann E., Rother C. (2018). iPose: Instance-Aware 6D Pose Estimation of Partly Occluded Objects. arXiv.

[9] He Z., Chavan-Dafle N., Huh J., Song S., Isler V. (2023). Pick2Place: Task-aware 6DoF Grasp Estimation via Object-Centric Perspective Affordance. arXiv.

[10] Zeng A., Song S., Yu K.-T., Donlon E., Hogan F.R., Bauza M., Ma D., Taylor O., Liu M., Romo E. (2022). Robotic Pick-and-Place of Novel Objects in Clutter with Multi-Affordance Grasping and Cross-Domain Image Matching. Int. J. Robot. Res..

[11] Song D., Park Y., Jo M., Hwang W., Yi S.J. Vision Based Pick and Place of Randomly Stacked Jenga Blocks Using a Single RGB-D Sensor. Proceedings of the International Conference on Ubiquitous Robots (UR).

[12] Nieuwenhuisen M., Droeschel D., Holz D., Stückler J., Berner A., Li J., Klein R., Behnke S. Mobile bin picking with an anthropomorphic service robot. Proceedings of the 2013 IEEE International Conference on Robotics and Automation.

[13] Holz D., Nieuwenhuisen M., Droeschel D., Stückler J., Berner A., Li J., Klein R., Behnke S. (2013). Active Recognition and Manipulation for Mobile Robot Bin Picking. Springer Tracts in Advanced Robotics.

[14] Liu H., Philipose M., Sun M.T. (2014). Automatic objects segmentation with RGB-D cameras. J. Vis. Commun. Image Represent..

[15] Toscana G., Rosa S., Bona B., Bi Y., Kapoor S., Bhatia R. (2018). Fast Graph-Based Object Segmentation for RGB-D Images. Proceedings of SAI Intelligent Systems Conference (IntelliSys) 2016.

[16] Herzog A., Pastor P., Kalakrishnan M., Righetti L., Bohg J., Asfour T., Schaal S. (2014). Learning of Grasp Selection Based on Shape-Templates. Auton. Robot..

[17] Xiang Y., Schmidt T., Narayanan V., Fox D. (2018). PoseCNN: A Convolutional Neural Network for 6D Object Pose Estimation in Cluttered Scenes. arXiv.

[18] He K., Gkioxari G., Dollár P., Girshick R. Mask R-CNN. Proceedings of the IEEE International Conference on Computer Vision (ICCV).

[19] Xu Y., Arai S., Liu D., Lin F., Kosuge K. (2022). FPCC: Fast point cloud clustering-based instance segmentation for industrial bin-picking. Neurocomputing.

